# Cu_2_O Nanoparticles as Nanocarriers and Its Antibacterial Efficacy

**DOI:** 10.3390/ph17091124

**Published:** 2024-08-26

**Authors:** María Isabel Torres-Ramos, Ubaldo de Jesús Martín-Camacho, Jorge Alberto Sánchez-Burgos, Suresh Ghotekar, Oscar Arturo González-Vargas, Mamoun Fellah, Alejandro Pérez-Larios

**Affiliations:** 1 Nanomaterials, Water and Energy Research Laboratory, Centro de Estudios para la Agricultura, la Alimentación y la Crisis Climática (CEAACC), Centro Universitario de los Altos, Universidad de Guadalajara, Tepatitlán de Morelos 47600, Mexico; isabel.torres7734@alumnos.udg.mx (M.I.T.-R.); ubaldo.martin2609@alumnos.udg.mx (U.d.J.M.-C.); 2 Integral Food Research Laboratory, National Technological of Mexico/Technological Institute of Tepic, Tepic 63175, Mexico; jsanchezb@tepic.tecnm.mx; 3 Centre for Herbal Pharmacology and Environmental Sustainability, Chettinad Hospital and Research Institute, Chettinad Academy of Research and Education, Kelambakkam 603103, India; ghotekarsuresh7@gmail.com; 4 Departamento de Ingeniería en Control y Automatización, ESIME-Zacatenco, Instituto Politécnico Nacional, UPALM, Av. Politécnico s/n, Col., Zacatenco, Alcadía Gustavo A. Madero, Ciudad de México 07738, Mexico; ogonzalezv@ipn.mx; 5 Mechanical Engineering Department, ABBES Laghrour University, P.O. Box 1252, Khenchela 40004, Algeria; mamoune.fellah@univ-khenchela.dz

**Keywords:** Cu_2_O nanoparticles, sol–gel method, drug delivery, functionalization, mesoporous materials

## Abstract

In this study, Cu_2_O nanoparticles were synthesized using the sol–gel technique and subsequently functionalized with extracts from plants of the Rauvolfioideae subfamily and citrus fruits. Comprehensive characterization techniques, including UV-Vis spectroscopy, FT-IR, XRD, BET, SEM, and TEM, were employed to evaluate the structural and surface properties of the synthesized nanoparticles. The results demonstrated that both functionalized Cu_2_O nanoparticles exhibit mesoporous structures, as confirmed by nitrogen adsorption–desorption isotherms and the pore size distribution analysis. The green extract functionalized nanoparticles displayed a more uniform pore size distribution compared to those functionalized with the orange extract. The study underscores the potential of these functionalized Cu_2_O nanoparticles for applications in drug delivery, catalysis, and adsorption processes, highlighting the influence of the functionalization method on their textural properties and performance in antibacterial efficacy.

## 1. Introduction

The growing resistance of bacteria to traditional antibiotics has driven the search for new therapeutic strategies [1]. Among these, the controlled release of natural extracts via nanoparticles has emerged as a promising solution [2,3,4]. Cuprous oxide (Cu_2_O) nanoparticles have garnered significant attention due to their unique properties, such as intrinsic antimicrobial activity, biocompatibility, and potential for the sustained release of bioactive compounds [5,6].

Natural extracts, with their multiple bioactive compounds, offer an attractive alternative to synthetic antibiotics [7,8]. However, their efficacy can be limited by low solubility, stability, and bioavailability [9]. Incorporating these extracts into controlled release systems can significantly enhance their therapeutic activity by protecting them from degradation and allowing for sustained release at the site of action [10,11,12].

In this context, Cu_2_O nanoparticles represent an innovative platform for the delivery of natural extracts. Their ability to interact with bacterial membranes, combined with the gradual release of bioactive compounds, can enhance the antibacterial efficacy of natural extracts [13,14]. Additionally, the use of Cu_2_O allows for the exploration of synergies between the inherent antimicrobial properties of cuprous oxide and the therapeutic effects of natural extracts [15].

This study aims to evaluate the efficiency of Cu_2_O nanoparticles as vehicles for the controlled release of natural extracts and determine their antibacterial efficacy. Through a series of experimental assays, the capacity of these nanoparticles to improve the stability and bioavailability of natural extracts, as well as their impact on bacterial growth inhibition, will be investigated.

The research presented herein seeks not only to advance the understanding of Cu_2_O nanoparticles’ applications in drug delivery but also to provide a foundation for the development of new antibacterial therapies based on natural extracts, thereby contributing to the fight against bacterial resistance.

## 2. Results and Discussion

**Figure 1:**
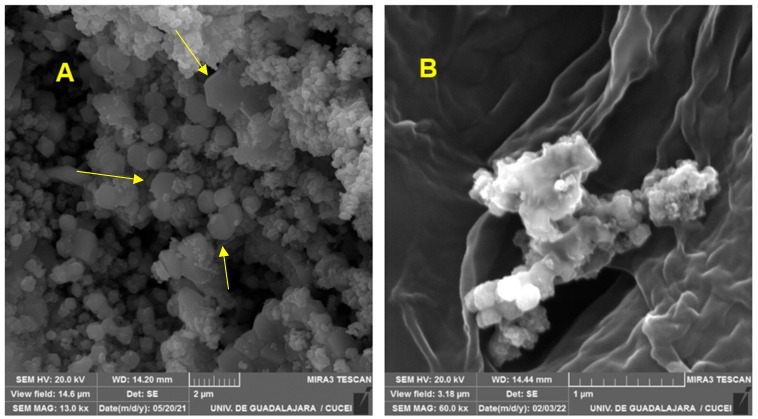
Cu_2_O micrographs. (**A**) Cu_2_O—N nanoparticles. (**B**) Functionalized material.

Figure 1 shows the micrographs of Cu_2_O. Figure 1A presents hexagon-shaped structures, which are characteristic of this material. The same morphologies can be seen under the polymeric coating applied in the functionalization, as presented in Figure 1B [16,17,18].

**Figure 2:**
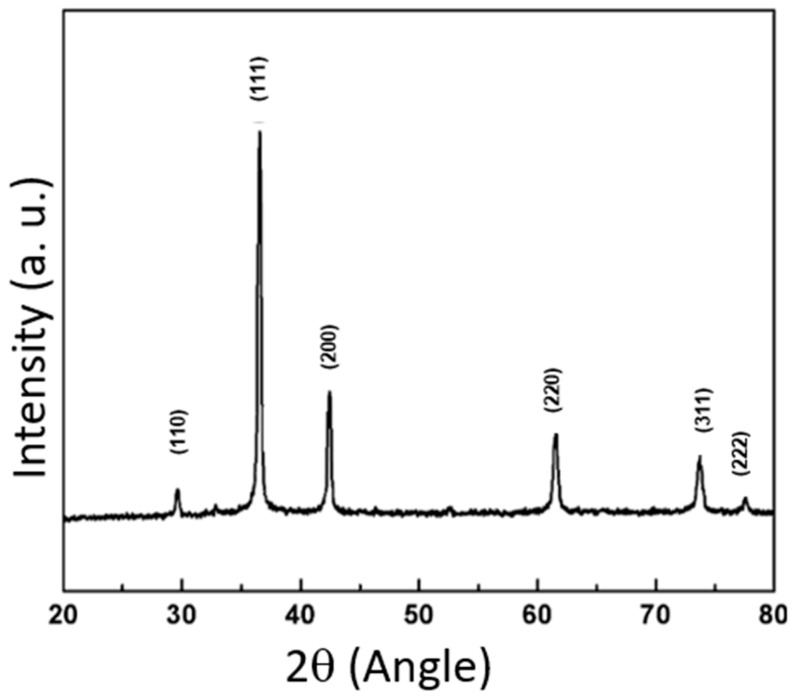
X-ray diffraction patterns of Cu_2_O.

In Figure 2, the X-ray diffraction (XRD) patterns of bare Cu_2_O oxide are presented. The observed diffraction peaks correspond to the crystallographic planes (110), (111), (200), (220), (311), and (222), confirming the presence of Cu_2_O in its face-centered cubic (FCC) phase [19]. The most intense peak, corresponding to the (111) plane, indicates a possible preferential orientation of the crystals in that direction [20]. The positions and relative intensities of the peaks are consistent with the expected values for Cu_2_O in this crystal structure [13,15,21].

The crystallite size, D, of the samples was estimated from the half width (b) of the peak at 2θ = 36° by the Scherrer formula: D = Kk/(b cos h). A crystallite size in the nanometer range (7.2–5.7 nm) was obtained in the sample [22].

The most intense peak observed at approximately 2θ ≈ 36°, corresponding to the (111) plane, suggests that the Cu_2_O nanoparticles have a preferential orientation in that direction [23]. This preferential orientation can influence the physical and chemical properties of the material, including its photocatalytic and antimicrobial activity. For instance, a greater exposure of certain crystallographic planes can enhance the material’s interaction with biological or catalytic agents [5,24].

**Figure 3:**
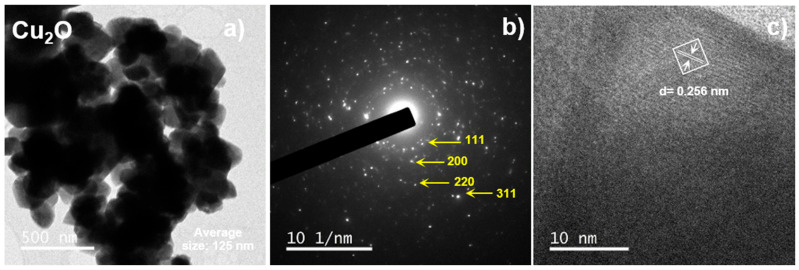
TEM Analysis of Cu_2_O. (**a**) Cu_2_O NP´s, (**b**) Cu_2_O SAED analysis and (**c**) Cu_2_O interplanar distance.

Figure 3 shows a cluster of Cu_2_O nanoparticles. The nanoparticles have an average size of 125 nm. The nanoparticles exhibit an irregular shape and appear to be agglomerated. The image provides an overview of the distribution and size of the nanoparticles [21,25].

TEM images provide a detailed characterization of Cu_2_O nanoparticles. The image (Figure 3a) confirms the size and morphology of the nanoparticles, showing a tendency to agglomerate, which is common in nanoparticles due to their high surface energy [26,27,28].

The electron diffraction pattern (Figure 3b) confirms the crystalline structure of the nanoparticles, corroborating the face-centered cubic (FCC) phase of Cu_2_O, consistent with the results obtained from the X-ray diffraction (XRD) [6,29]. The presence of well-defined rings indicates a high crystallinity of the nanoparticles [30].

The HRTEM analysis (Figure 3c) provides an accurate measurement of the interplanar distances, which are important characteristics for identifying the crystalline structure [31]. The distance of 0.256 nm corresponds to the interplanar spacings of the (111) planes in the Cu_2_O structure, further supporting the face-centered cubic phase [32,33,34].

Together, these samples confirm that the synthesized Cu_2_O nanoparticles have a well-defined crystalline structure and morphological properties that may be suitable for applications in controlled drug release and antimicrobial activities.

**Figure 4:**
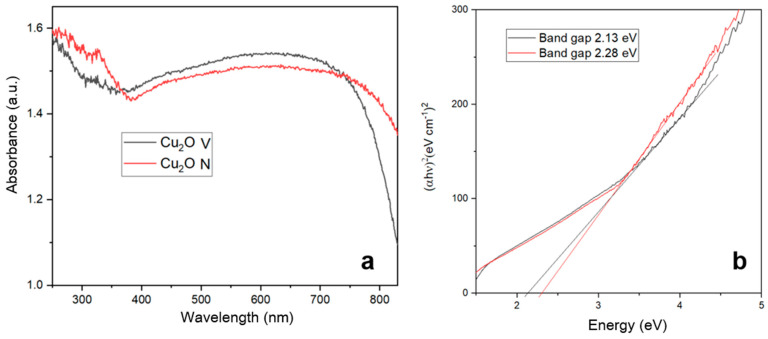
Spectrum of UV-Vis of Cu_2_O. (**a**) UV-vis absorption and (**b**) band gap energies.

Figure 4 shows the optical properties of Cu_2_O nanoparticles. In Figure 4a, the nanoparticles were measured over a wavelength range of 200 to 900 nm. Both samples exhibit a strong absorbance in the UV-visible region, with a significant absorption below 600 nm. Cu_2_O-V shows a slightly higher absorbance at lower wavelengths (around 200–300 nm) compared to Cu_2_O-N. The absorbance decreases as the wavelength increases for both samples, indicating a typical semiconductor absorption behavior [23,24]. The differences in absorbance between Cu_2_O-V and Cu_2_O-N might be due to variations in particle size, crystallinity, or surface properties [35]. In the other hand, Figure 4b shows the Tauc plots used to determine the band gap energies of Cu_2_O-V and Cu_2_O-N. The plot displays (αhv)^2^(versus energy (eV). The band gap for Cu_2_O-V (black line) is determined to be 2.13 eV, and for Cu_2_O N (red line), it is determined to be 2.28 eV [36]. The band gap energies are slightly different between the two samples, suggesting variations in their electronic structure. The slight difference in band gap energies might be attributed to changes in particle size, defect states, or doping levels [37,38]. A higher band gap for Cu_2_O-N indicates it might be more effective in applications requiring higher energy photons [39].

**Figure 5:**
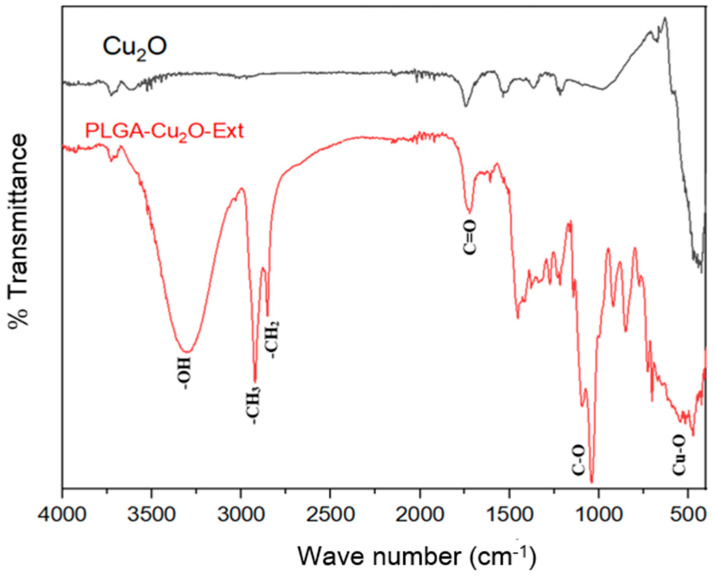
FTIR spectra comparison of pure Cu_2_O and PLGA-coated Cu_2_O NPs with natural extract.

Figure 5 shows the Fourier transform infrared spectroscopy (FTIR) spectra for two samples: Cu_2_O and PLGA-Cu_2_O-Ext. The *y*-axis represents the percentage transmittance, while the *x*-axis represents the wavenumber in cm^−1^, which corresponds to different vibrational modes of the molecules.

The FTIR spectrum of Cu_2_O shows distinct peaks that correspond to various vibrational modes associated with the material [21]. Around 600 cm^−1^, this peak can be attributed to the Cu-O stretching vibration, characteristic of Cu_2_O [40]. The spectrum does not show significant peaks in the higher wavenumber region, which is expected for pure Cu_2_O, as it lacks organic functional groups [28].

The PLGA-Cu_2_O-Ext spectrum displays additional peaks, indicating the presence of organic functional groups and interactions between the components. At around 3500–3200 cm^−1^, a broad peak corresponding to O-H stretching vibrations indicates the presence of hydroxyl groups. At around 3000–2800 cm^−1^, peaks corresponding to C-H stretching vibrations from -CH_3_ and -CH_2_ groups suggest the presence of aliphatic chains [41,42]. At around 1750–1700 cm^−1^, a peak corresponding to C=O stretching vibrations is observed, which is characteristic of carbonyl groups found in PLGA [43]. At around 1250–1000 cm^−1^, peaks corresponding to C-O stretching vibrations indicate ester functionalities in PLGA. At around 600 cm^−1^ [44], the presence of Cu-O stretching vibrations are indicated.

**Figure 6:**
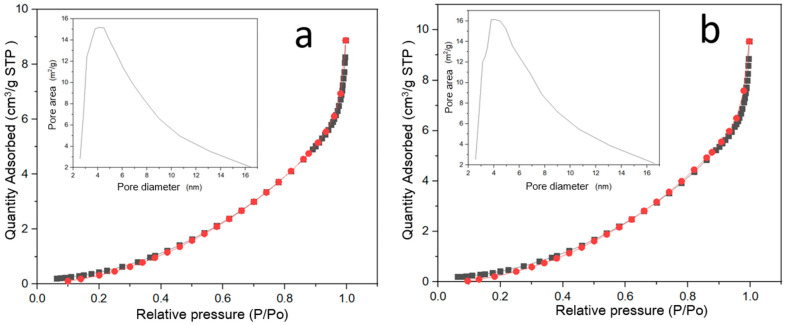
Nitrogen adsorption–desorption isotherms and pore size distribution of Cu_2_O nanoparticles functionalized with natural extracts, (**a**) green extract (Cu_2_O-VE) and (**b**) orange extract (Cu_2_O-NE).

Figure 6 shows nitrogen adsorption–desorption isotherms and corresponding pore size distribution plots (insets) for Cu_2_O nanoparticles functionalized with two different extracts, labeled as (a) green extract (Cu_2_O-VE) and (b) orange extract (Cu_2_O-NE).

Both isotherms exhibit a typical type IV shape according to the IUPAC classification, indicating the presence of mesoporous structures. The hysteresis loops observed at higher relative pressures (P/Po) are characteristic of capillary condensation in mesopores, suggesting the material’s mesoporous nature. The amount of nitrogen adsorbed increases with relative pressure, indicating the progressive filling of mesopores.

The pore size distribution for both samples shows a significant peak in the mesopore range, around 4–10 nm. The peak height and width provide information about the pore volume and uniformity of the pore sizes. The Cu_2_O-VE sample (Figure 6a) has a slightly narrower and more pronounced peak compared to the Cu_2_O-NE sample (Figure 6b), suggesting a more uniform pore size distribution for the green extract functionalized nanoparticles.

**Figure 7:**
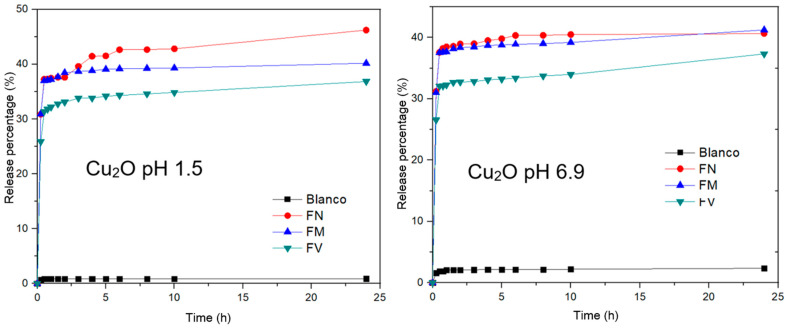
Release profiles of natural extracts from Cu_2_O NPs at different pH.

Figure 7 shows the release profiles of natural extracts from Cu_2_O nanoparticles at two different pH levels: 1.5 and 6.9. The figure with Cu_2_O pH 1.5 shows a control sample with a negligible release. The FN sample shows the highest release rate, reaching about 45%, the FM sample shows a moderate release rate, reaching around 35%, and the FV sample shows the lowest release rate among the experimental samples, reaching around 30% in 24 h.

The highest release rate reaches about 45%. The figure with Cu_2_O pH 6.9 shows a moderate release rate, reaching around 35%, and this sample shows the lowest release rate among the experimental samples, reaching around 30%; all delivery samples were released in 24 h.

All samples (except the control) exhibit a rapid initial release within the first 5 h, followed by a slower, sustained release up to 24 h. The variation in release rates among FN, FM, and FV suggests different interactions or affinities between the natural extracts and the Cu_2_O matrix.

The figure with Cu_2_O pH 6.9 shows a control sample with a negligible release, similar to the acidic pH condition. The FN sample shows a high release rate, reaching about 40%, the FM sample shows a moderate release rate, reaching around 35%, and the FV sample shows a lower release rate, reaching around 30%; all delivery samples were released in 24 h.

Similar to the acidic condition, all experimental samples show a rapid initial release, followed by a slower release. The release profiles are relatively consistent between the acidic and neutral pH levels, indicating that the Cu_2_O nanoparticles are stable and effective in controlling the release of natural extracts across a range of pH levels [45,46].

An evaluation was carried out to determine the MIC and MBC of each of the functionalized materials and the results are presented below and distributed in tables. It is worth remembering that for each material, there are three functionalizations: the FN sample with the orange extract, the FM sample, which involved the mixture of the green extract and orange in equal parts, and the FV sample, which only used green extract. The results of this last treatment will not be reflected in the tables since it did not present antibacterial activity in any of the materials, which is supported by other authors who attribute properties such as anti-inflammatory, neuroprotective, and even cognitive enhancing activities to the alkaloids; however, they do not have sufficient antibacterial or antifungal activity to be considered antimicrobial agents.

Table 1 lists the Minimum Inhibitory Concentrations (MICs) and Minimum Bactericidal Concentrations (MBCs) of two types of functionalized nanoparticles (FM and FN) against five bacterial species: *Listeria monocytogenes*, *Enterococcus faecalis*, *Staphylococcus aureus*, *Salmonella paratyphi*, and *Escherichia coli*. These measurements are crucial in determining the antibacterial efficacy of the nanoparticles, where the MIC indicates the lowest concentration that prevents the visible growth of a bacterium, and the MBC represents the lowest concentration at which the nanoparticles kill the bacteria.

### 2.1. Functionalized FM Nanoparticles

A variable efficacy is shown across the bacterial strains. The functionalized FM nanoparticles are notably effective against *Salmonella paratyphi* and *Escherichia coli*, with an MIC of 40 µg/mL and 80 µg/mL, respectively, and corresponding MBC values indicating a bactericidal effect at significantly higher concentrations. The functionalized FM nanoparticles exhibit a high bactericidal concentration for *Staphylococcus aureus* (640 µg/mL), suggesting a lower efficacy in completely killing the bacteria as compared to inhibiting its growth.

### 2.2. Functionalized FN Nanoparticles 

Generally more effective than the FM sample, the FN sample shows lower MIC values for *Listeria monocytogenes* and *Salmonella paratyphi* (20 µg/mL). There are consistent MBC values for *Enterococcus faecalis* and relatively lower MBC values for other strains compared to the FM sample, indicating a more potent bactericidal capability.

The varying responses of different bacteria to the same nanoparticles suggest that the mechanism of action could be influenced by the bacterial cell wall composition and the metabolic pathways of each species. For instance, Gram-positive bacteria like *Staphylococcus aureus* exhibit a higher resistance to the bactericidal action of FM nanoparticles. FN nanoparticles demonstrate a more consistent and effective antibacterial activity both in terms of inhibition and bactericidal action across all tested bacteria. This could be attributed to differences in the surface properties, size, or the concentration of the active component in the FN formulation compared to the FM formulation. The data suggest that FN nanoparticles might be more suitable for broader applications in treating or preventing bacterial infections due to their lower MIC and MBC values. However, the specific application would need to consider the target bacterial infection and the local environment, as the effectiveness can vary. The relatively high concentrations needed for bactericidal action (especially noted in FM for *Staphylococcus aureus*) could contribute to the development of resistance. Continued monitoring and combination strategies might be necessary to mitigate such risks.

This visual representation underscores the importance of determining the optimal concentrations and formulations for maximizing antibacterial activity while minimizing the risk of bacterial resistance development. Future studies should focus on understanding the mechanisms of action and further optimizing the nanoparticle formulations for specific applications.

## 3. Materials and Methods

Some characteristics of the extracts are protected because they are in the process of international patent approval with application No. PCT/IB2020/061916.

### 3.1. Synthesis of Nanoparticles

The materials of Cu_2_O were prepared using a modified sol–gel synthesis [47]. Copper (II) nitrate was used as a precursor (reagents obtained from Sigma-Aldrich Chemical Co., St. Louis, MO, USA). A total of 40 mL of extract was placed in a three-neck flask, and the precursor was added dropwise, maintaining a temperature of 70 °C with constant stirring and under a vapor condensation system for 4 h. Subsequently, the solution was transferred to a jacketed reactor at 0 °C with stirring for 20 h. The resulting xerogel was dried at 60 °C, and the solid was ground in an agate mortar and then calcined at 500 °C for 5 h in a static air atmosphere with a heating rate of 2 °C/min.

### 3.2. Functionalization of Nanoparticles

The nanoparticles (NPs) were functionalized using an organic phase containing ~5 mg of Poly(D,L-lactide-co-glycolide) (PLGA) (75:25) and 400 µL of acetone, and an aqueous phase composed of 5 mL of a poly-vinyl acetate (PVA) solution (4%) and extracts (16% *w*/*v*). To this, 5 mg of nanomaterial was added and sonicated for 3 min. Subsequently, the phases were homogenized using an Ultra-turrax device (IKA, T18; Wilmington, NC, USA), with the organic phase added dropwise. The resulting samples were then maintained at −80 °C for 2 h and lyophilized at −50 °C (Labconco, FreeZone 6; Kansas, MO, USA) [48].

### 3.3. Characterization of Nanoparticles

Once the nanoparticles were obtained, it was necessary to determine their structural and surface characteristics. The size and distribution affect various properties and their potential applications; hence, it is essential to determine at least two main parameters once the nanoparticles are obtained.

The commonly used techniques to determine the structural and surface details of the synthesized nanoparticles include the following: UV-Vis spectroscopy, Fourier transform infrared spectroscopy (FT-IR), X-ray diffraction (XRD), nitrogen physisorption (BET), and scanning electron microscopy (SEM).

#### 3.3.1. Scanning Electron Microscopy (SEM)

The morphology of the materials was observed using a scanning electron microscope (MIRA 3LMU, Tescan, London, UK), which was operated at 20 kV.

#### 3.3.2. Transmission Electron Microscopy (TEM)

High-resolution images were obtained using a transmission electron microscopy (Jeol, JEM ARM200F, Boston, MA, USA) operated at 200 kV, and the images were analyzed using specialized software (Gatan Micrograph v. 3.7.0, Gatan Inc., Pleasanton, CA, USA).

#### 3.3.3. X-ray Diffraction (XRD)

Powder X-ray diffraction patterns were acquired using a Panalytical XRD diffractometer (Empyrean, Almelo, The Netherlands) equipped with Cu Kα radiation (λ = 0.154 nm). Data were collected from 10° to 90° (2θ) with a scanning speed of 0.02°/0.2 s.

#### 3.3.4. Nitrogen Physisorption (BET Method)

The textural properties (surface area, pore volume, and pore size) were determined by nitrogen adsorption-desorption with a Micromeritics (TriStar II Plus, Norcross, GA, USA). The samples were degassed at 200 °C for 2 h under a vacuum. Nitrogen adsorption isotherms were measured at liquid nitrogen temperature (77 K) with nitrogen pressures ranging from 10^–6^ to 1.0 P/P_0_. The specific surface area was obtained by the Brunauer–Emett–Teller method (BET) and the pore size distribution following the Barret–Joyner–Halenda (BJH) method.

#### 3.3.5. UV-Vis Spectroscopy

The absorption spectra of the materials were acquired using a Shimadzu UV-Vis spectrophotometer (UV-2600, Tokyo, Japan), which was equipped with an integrating sphere suitable for diffuse reflectance studies. UV-Vis spectra were analyzed at wavelengths from 190 to 900 nm [49].

The band-gap energy was calculated using the Tauc Plot method, as shown in Equation (1):

(1) $${\left(\alpha hv\right)}^{\gamma }=A(hv-E_{g})$$

where α is the absorption coefficient, h is the Planck’s constant, v is the photon frequency, A is a proportionality constant determined by the refractive index, Eg is the band-gap energy, and the exponent γ denotes the nature of the electronic transition (γ = 2 for a direct transition, γ = 1/2 for an indirect transition).

#### 3.3.6. Fourier Transform Infrared Spectroscopy (FT-IR)

The FTIR spectra for the material were recorded with an FTIR (Thermo Fisher Scientific, Nicolet iS5, Tokyo, Japan) spectrophotometer using attenuated total reflectance (ATR) with a diamond waveguide (XR model). A detector of fast recovery deuterated triglycine sulfate (DTGS) (standard) was used for the analysis. The spectra were recorded at room temperature, with 24 scans and 4 cm^−1^ of resolution. Samples were recorded from 4000 cm^−1^ to 400 cm^−1^.

### 3.4. Release Profile of Extracts in the NP

The in vitro release profile of the extracts was evaluated using a dialysis tube. First, 5 mg of nanoparticles were dissolved in 5 mL of buffer (pH = 1.5 and pH = 7.0) inside a dialysis tube. This tube was immersed in 40 mL of buffer (pH = 1.5 and pH = 7.0) at 37 °C with stirring at approximately 100 RPM, serving as the release medium. Samples (2 mL) were taken at 15, 30, 45, 60, 90, 120, 180, 240, 300, 360, 420, 480, 540, 600, and 1440 min, replacing the same volume of fresh buffer. Absorbance readings were taken using a UV-Vis spectrophotometer (Shimadzu UV-2600, Tokyo, Japan) at 210 and 256 nm, and concentrations were calculated using the calibration curve equation for the natural extract (r = 0.99).

### 3.5. Bacterial Inhibition Assay

#### 3.5.1. Minimum Inhibitory Concentration (MIC)

Gram-negative bacterial strains (*Salmonella paratyphi* ATCC 9150 and *Escherichia coli* ATCC 8739) and Gram-positive strains (*Staphylococcus aureus* ATCC 33862, *Listeria monocytogenes* ATCC 15313, and *Enterococcus faecalis* ATCC 19433) were aerobically cultured in Mueller–Hinton broth (21 g/L, pH 7.3 ± 0.1) for 24 h at 37 °C. Bacterial suspensions were adjusted to a concentration of 1 × 10^6^ CFU/mL according to the 0.5 McFarland standard. Dilutions were made in Mueller–Hinton broth with a concentration range of 1280 to 2.5 µg/mL of nanomaterial, and the bacterial inoculum was added to the treatments. Incubation was performed for 24 h at 37 °C.

#### 3.5.2. Minimum Bactericidal Concentration (MBC)

To determine the MBC, 500 µL of the treatments resulting from the MIC were plated using the agar diffusion method on Mueller–Hinton agar plates with a concentration of 37 g/L and a pH of 7.3 ± 0.1, with dimensions of 100 × 15 mm in diameter. Plates were incubated for 24 h at 37 °C.

## 4. Conclusions

The synthesis and functionalization of nanoparticles via the sol–gel technique proved effective for Cu_2_O. By utilizing specific precursors and maintaining controlled reaction conditions, we successfully produced nanoparticles with the desired properties. The functionalization process, involving PLGA and PVA, allowed for the creation of stable nanomaterial systems, potentially enhancing their applicability in various fields.

Characterization techniques, such as UV-Vis spectroscopy, FT-IR, XRD, BET, SEM, and TEM, were crucial in determining the structural and surface properties of the synthesized nanoparticles. These methods provided detailed insights into the morphology, size distribution, surface chemistry, and crystalline structure, which are essential for understanding their behavior and potential applications.

SEM and TEM analyses revealed the morphology and detailed structure of the nanoparticles, while the XRD analysis confirmed their crystalline phases. The FT-IR spectroscopy analysis identified the presence of functional groups, indicating a successful surface modification. The UV-Vis spectroscopy and Tauc Plot analysis provided information about the optical properties and band-gap energies of the nanoparticles, which is crucial for applications in photocatalysis and other fields.

The provided MIC and MBC values highlight the potential of these functionalized nanoparticles as antibacterial agents against a spectrum of pathogenic bacteria. The consistent performance of the FN formulation suggests its suitability for further development and potential clinical applications. However, the variability in effectiveness underscores the need for tailored approaches depending on the specific bacterial strain and the clinical scenario. Future studies should explore the mechanisms of action to enhance the efficacy and reduce the potential for resistance.

## Figures and Tables

**Table 1:** Comparative analysis of MICs and MBCs of functionalized NP’s against various pathogenic bacteria.

	*Listeria monocytogenes*		*Enterococcus faecalis*		*Staphylococcus aureus*		*Salmonella paratyphi*		*Escherichia coli*	
	MIC	MBC	MIC	MBC	MIC	MBC	MIC	MBC	MIC	MBC
*FM*	80	160	40	60	40	640	40	640	80	320
*FN*	20	80	40	60	40	320	20	160	40	160

## Data Availability

The original contributions presented in the study are included in the article, further inquiries can be directed to the corresponding author.
